# Metamaterial design for aortic aneurysm simulation using 3D printing

**DOI:** 10.1186/s41205-024-00219-w

**Published:** 2024-08-07

**Authors:** Arthur K. F. Sakai, Ismar N. Cestari, Eraldo de Sales, Marcelo Mazzetto, Idágene A. Cestari

**Affiliations:** 1 Electrical Engineering Graduate Program, Telecommunications and Control Engineering Department, Polytechnic School, University of São Paulo, São Paulo, Brazil; 2 Laboratório de Bioengenharia, Instituto do Coração, Hospital das Clínicas HCFMUSP, Faculdade de Medicina, Universidade de São Paulo, São Paulo, Brazil

**Keywords:** 3D printing, Metamaterials, Biomechanics, Aortic aneurysm

## Abstract

**Introduction:**

The use of three-dimensional (3D) printed anatomic models is steadily increasing in research and as a tool for clinical decision-making. The mechanical properties of polymers and metamaterials were investigated to evaluate their application in mimicking the biomechanics of the aortic vessel wall.

**Methodology:**

Uniaxial tensile tests were performed to determine the elastic modulus, mechanical stress, and strain of 3D printed samples. We used a combination of materials, designed to mimic biological tissues’ properties, the rigid Vero^TM^ family, and the flexible Agilus30™. Metamaterials were designed by tessellating unit cells that were used as lattice-reinforcement to tune their mechanical properties. The lattice-reinforcements were based on two groups of patterns, mainly responding to the movement between links/threads (chain and knitted) or to deformation (origami and diamond crystal). The mechanical properties of the printed materials were compared with the characteristics of healthy and aneurysmal aortas.

**Results:**

Uniaxial tensile tests showed that the use of a lattice-reinforcement increased rigidity and may increase the maximum stress generated. The pattern and material of the lattice-reinforcement may increase or reduce the strain at maximum stress, which is also affected by the base material used. Printed samples showed max stress ranging from 0.39 ± 0.01 MPa to 0.88 ± 0.02 MPa, and strain at max stress ranging from 70.44 ± 0.86% to 158.21 ± 8.99%. An example of an application was created by inserting a metamaterial designed as a lattice-reinforcement on a model of the aorta to simulate an abdominal aortic aneurysm.

**Conclusion:**

The maximum stresses obtained with the printed models were similar to those of aortic tissue reported in the literature, despite the fact that the models did not perfectly reproduce the biological tissue behavior.

## Introduction

### Use of 3D printing to create anatomic models

Applications of three-dimensional (3D) printing technology in medicine include education [1], training [2; 3], device innovation [4], surgical planning [5; 6] and stent fenestration [7], among others.

Stratasys® PolyJet™, which uses multimaterial printing technology, jets droplets of different photopolymers in liquid form from a printing header, and cures them with ultraviolet light. This technology allows specific regions to be printed using different materials and is useful in medical research applications [8; 9]. For instance, to mimic certain diseases, a flexible model of a blood vessel with rigid parts representing calcifications on the vessel wall can be printed using PolyJet™ [10]. This technology also allows the mixing of base polymers to create a material with intermediate characteristics, called digital materials [11].

Kaschwich et al. (2021) [12] used PolyJet™ technology to investigate the geometric accuracy of 3D printed models of abdominal aortic aneurysms using computed tomography (CT) images. The model was printed using a mixture of TangoPlus™ (flexible) and VeroClear™ (rigid) photopolymers. The resulting models were scanned on the same CT device and compared to the original images. Comparing the 3D printed models and the original images, the mean deviation ranged from − 0.73 mm to 0.14 mm, and the relative deviation showed no significant difference. The results showed that PolyJet™ technology can reliably reproduce vascular models with a high dimensional accuracy.

Tee et al. (2020) [13] tested the mechanical behavior of materials used in anatomic models on the J750™ Digital Anatomy™ Printer (DAP). The materials studied were the Agilus30™ (flexible) and the VeroMagentaV™ (rigid) photopolymers. In the uniaxial tensile tests, different models were tested: pure flexible, pure rigid, digital materials with different material proportions, and hybrids made of pure flexible with rigid particles in 5% volume and vice-versa. The results showed that the inclusion of rigid particles in the flexible material drastically reduced the strain at maximum stress while slightly reducing the maximum stress.

Lumpe et al. (2019) [14] studied the tensile properties of 3D printed multi-material interfaces that occurs when different materials are used in the same object, using the uniaxial tensile test. In all tested samples, one material at the interface was always the VeroWhitePlus™ (rigid), while three other materials were tested: pure TangoBlackPlus™ (flexible), and two digital materials. This study also tested how printing positioning affects the properties of the interface, because the printer moves the printing header in one direction at a time. The results show that the samples tend to fail at the interface if both materials that form it are rigid, but they tend to fail in the middle of the flexible material when one of them is more flexible. The results also show that the effect of printing positioning depends on the materials used.

Severseike et al. (2019) [9] tested the mechanical behavior of 3D printed digital materials, mixing Agilus30™ and TissueMatrix™ using PolyJet™ printer. These digital materials were developed by the manufacturer to mimic anatomical tissues. A model composed of pure Agilus30™ was compared with the porcine myocardium, regarding puncture strength, compliance, compliance repeatability, and suturing. The results showed that, although the printed models are not a perfect replication of the biological myocardium, they show promise for simulating the biological tissue with some adjustments. A qualitative assessment by expert reviewers in this study compared printed samples and biological tissue using suturing and cutting as criteria. The models printed with digital materials had a better performance than the pure Agilus30™.

Kwon et al. (2020) [15] tested a few patterned designs to mimic the mechanical behavior of aortic tissue, using multi-material 3D printing to create models with the pattern made of VeroCyan™ (rigid) printed inside Agilus30™. The results show that, although the maximum stress and strain at the maximum stress of the printed models could meet the values of the aortic tissue, there are differences in stress and modulus of elasticity that are dependent on the strain.

Cloonan et al. (2014) [16] demonstrated the applicability of 3D printing for idealized and patient-specific aortic models, comparing 3D printed and investment casted models, using various tests. The results show that the 3D printing is adequate for generation of biomedical phantoms, is a cost-efficient choice compared to the investment casting, and is capable of producing models of complex geometry and tortuosity with superior dimensional tolerances.

Wang, Zhao, et al. (2016) [17] and Wang, Wu, et al. (2016) [18] studied different patterns to reproduce the mechanical behavior of the soft biological tissue. Three-dimensional patterns made of the rigid material inside a matrix of the flexible material, using TangoPlus™ (flexible) and VeroBlack™ (rigid) as printing materials were tested. The results show strain-stiffening behavior, which is characteristic of soft tissues, but fine-tuning of the parameters is necessary to simulate real biological tissue.

Zhalmuratova (2019) [19] reported an application in device innovation, where the bi-dimensional patterns of a rigid material (VeroCyan™) inside a flexible matrix (Agilus30™) were created to obtain a pattern to be used in ex vivo perfusion devices. The intended material was one with strain-stiffening behavior at physiological strain values, and strain-softening behavior for larger strains. The former replicates the biological tissue, whereas the latter avoids negative consequences to the heart as the strain rises beyond physiological values.

This work reports the use of PolyJet™ technology, using its multimaterial printing capabilities to replicate the mechanical behavior of healthy and aneurysmal aortic tissue.

### Aorta and aortic aneurysm

In a simplified model, the aortic vessel wall is considered a viscoelastic material divided into three layers separated by elastic membranes [20]. The innermost layer, called the tunica intima, is formed by an endothelial layer and an internal elastic membrane, composed of elastin and collagen. The middle layer, called tunica media, is composed of elastin, collagen and smooth muscle cells, and takes the load at physiologically normal pressures. The outermost layer, called tunica adventitia, is composed mainly of helically positioned collagen fibers and is a load-bearing element at high pressures [20].

Aortic aneurysm is an aortic disease that causes >= 50% dilation of the expected diameter of a region of the aorta and can cause its rupture [21; 22], with approximately 20% chances of survival [23].

**Fig. 1:**
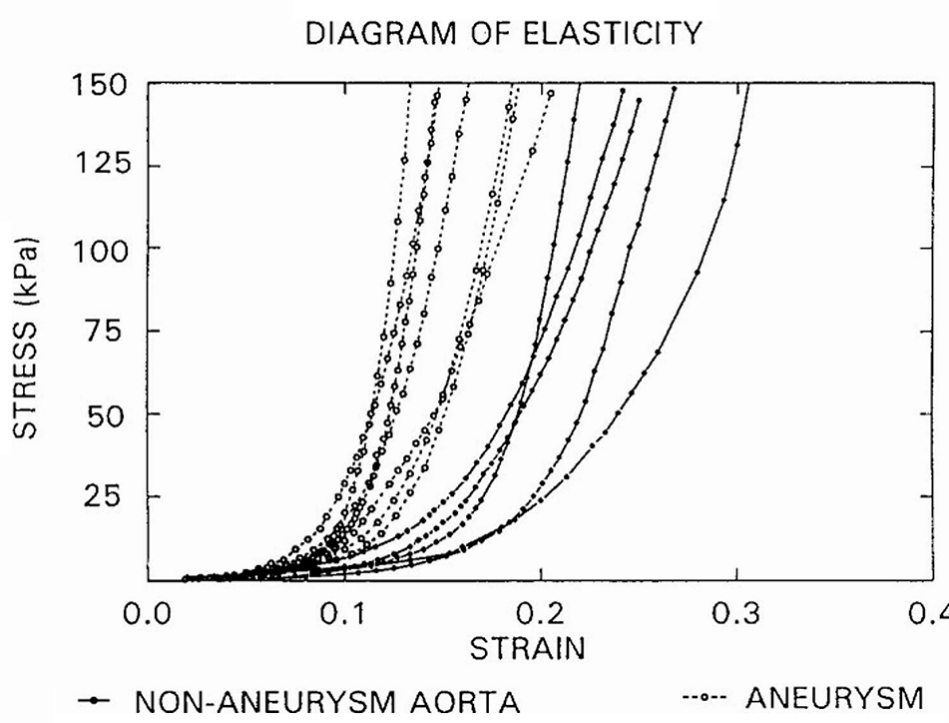
Stress-strain graph of normal (solid lines) and aneurysmal (dotted lines) aortic walls. Reproduced from He and Roach, (1994) [24] with permission from Elsevier

The aneurysm changes the mechanical behavior of the wall compared to that of a healthy aorta [24]. During stretching, the healthy aorta behaves like a soft tissue characterized by higher flexibility in smaller deformations and more stiffness as the tissue is stretched, a behavior known as strain-stiffening. In contrast, in aortic aneurysms, stiffening starts at smaller strains (Fig. 1). The exponential component of stress-strain response of the aortic wall is a result of the anisotropic material composition well described by the nonlinear material hyperelastic model proposed by Holzapfel, Gasser and Ogden (2000) [25].

### Metamaterials

**Fig. 2:**
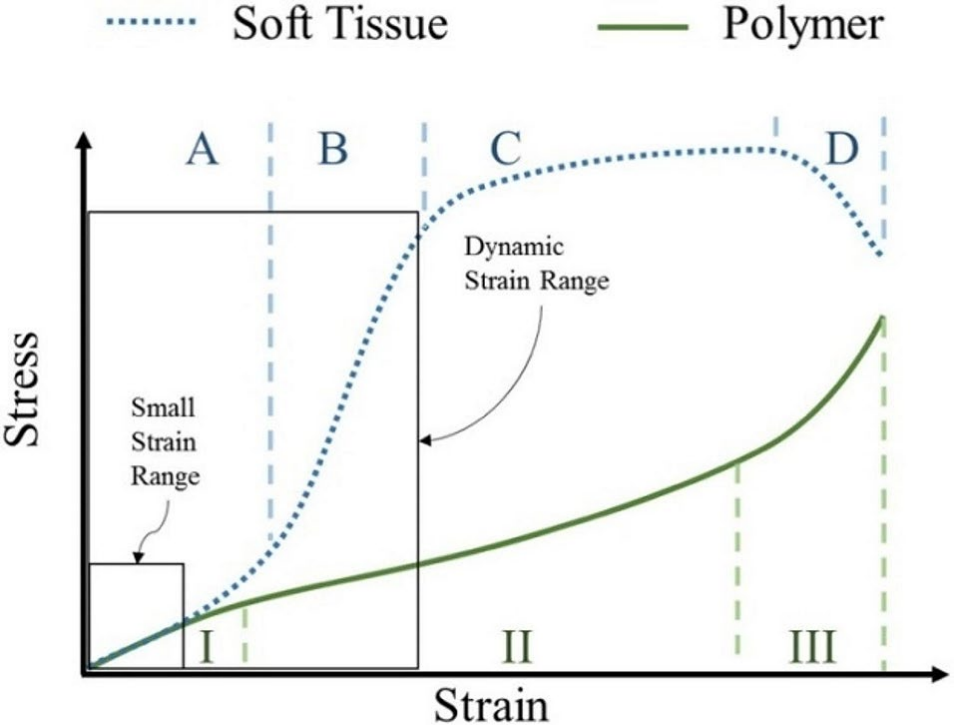
Typical stress-strain graph comparing soft tissue (dotted line) and polymer (solid line). Soft tissue: A, toe region; B, elastic region; C, plastic region; D, failure region. Polymer: I, primary creep; II, secondary creep; III, tertiary creep. Reproduced from Wang, Zhao, et al. (2016) [17] with permission from Elsevier

The mechanical behavior of soft biological tissues is different from that of polymeric 3D printing materials [17], as shown in Fig. 2. At strain levels up to 40% [26], the soft tissue and the polymeric material behave similarly. However, at higher strain levels, from 40 to 100% [26], the soft tissue becomes stiffer. This is shown by the larger increase in stress with increased strain, while the polymer maintains its elasticity, displayed by the smaller increase in stress with strain.

Metamaterials, materials whose structure affects their properties [18], were designed and used in this study. Kwon et al. (2020) [15], Wang, Zhao, et al. (2016) [17], Wang, Wu, et al., (2016) [18], and Zhalmuratova (2019) [19] investigated the use of metamaterials to modify the mechanical behavior of polymers.

This work investigated the mechanical properties of printing materials and metamaterials. Metamaterials were designed as lattice structures to be used as reinforcements inserted on a sample body of a different base material, resulting in a lattice-reinforcement (LR). These lattice structures, which are composed of repeated smaller elements called unit cells, can be created by tessellation of unit cells across a desired space [27].

## Methodology

### Printing materials

In this work, the following printing materials were used: VeroCyan™ (VCyan), VeroMagenta™ (VMgnt), Agilus30™ (A30), TissueMatrix™ (TMat), GelMatrix™ (GMat), BoneMatrix™ (BMat), and the support SUP706B™ [28]. The samples studied were printed on a J750™ DAP (Stratasys®, Rehovot, Israel) [29], a PolyJet™ printer capable of printing multiple materials simultaneously, with an accuracy of up to 100 μm, and a minimum layer thickness of 14 μm. Materials used in this study are listed in Table 1, along with their base materials and a short description.

**Table 1:** Printing materials used in this study

Material	Model Type	Base materials	Description
SH-Axx (SHAxx)	General	A30 + VCyan/VMgnt	Shore Hardness A xx
Tissue 600 (T600)	General	A30 + TMat	Simulates soft tissue
Slightly dense Bone (SDB)	Anatomy	NA	Simulates slightly dense bones

xx corresponds to the shore hardness of the material (30, 70, 85 or 95). NA – Not available

### Printed models

The printed models were designed with 3-matic® Medical software (version 17.0) [31]. Lattice microstructures were created, using Boolean operations and tessellating a unit cell, to be embedded as reinforcement in a flat rectangular sample [32].

In previous works, the chain [32] and knitted pattern [26] showed strain-stiffening behavior in uniaxial tensile tests. Their structures allowed free movement between the links/threads for smaller strains, and at larger strains, the links/threads act to transfer the stress between each other, creating the strain-stiffening behavior. The origami [33] and the diamond crystal patterns were expected to show a strain-stiffening behavior. The beams that form these patterns were expected to align along the tensile directions as the whole pattern stretches due to straining, and, as the beams become more aligned, the continuous straining would start applying tensile stress on the beams, resulting in a strain-stiffening behavior.

**Fig. 3:**
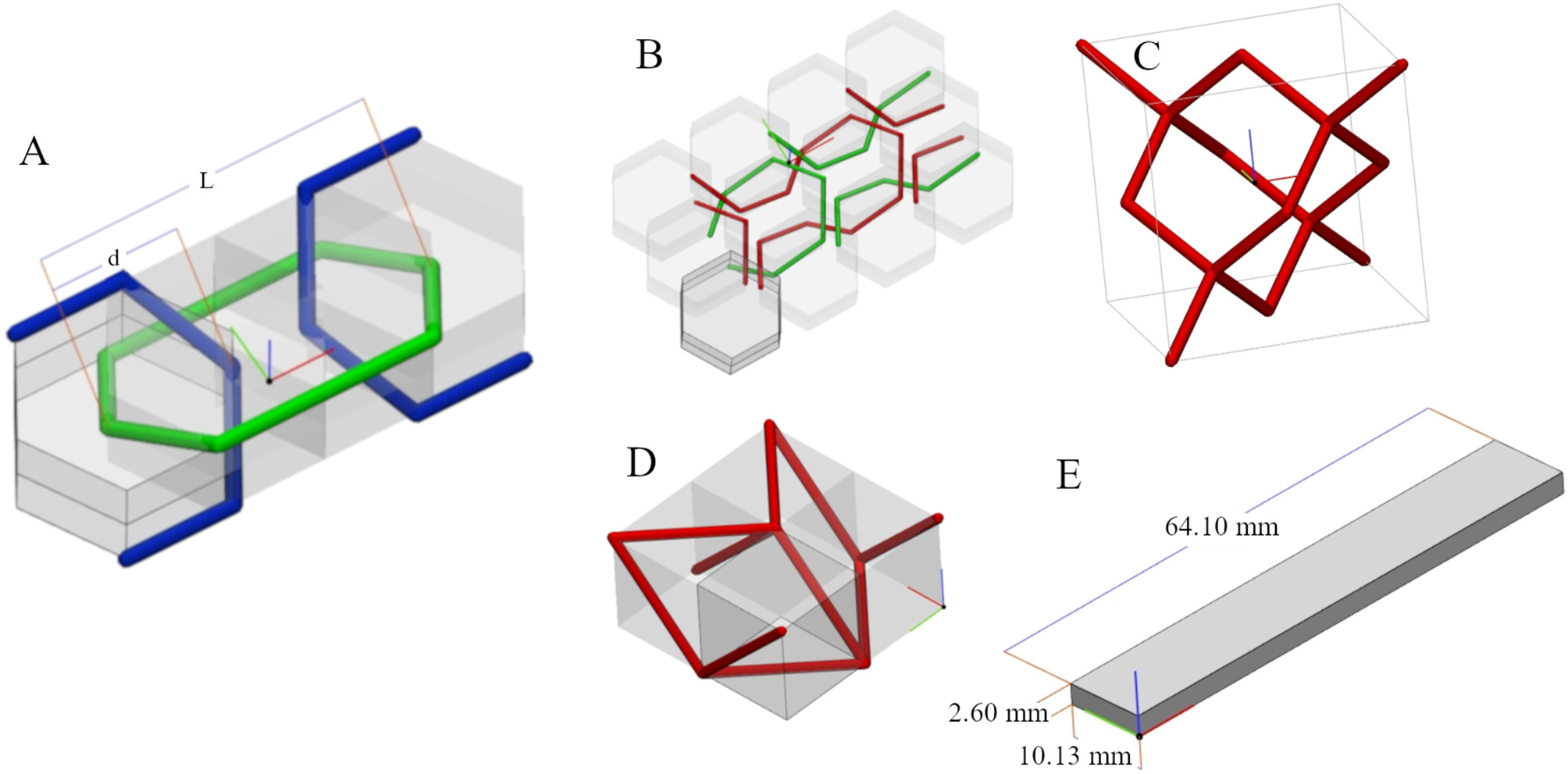
Schematic representation of the basic geometric forms of the unit cells (A) chain, (B) knitted pattern, (C) diamond crystal, (D) origami, and the retangular shaped base material (E). In (A) the distance d between the links was set to one-third of the lenght L.

The chain and knitted patterns were created from regular hexagonal prisms with a radius of 5 mm and a height of 5 mm, marked at a height of 2.5 mm, and the origami was created from cubes with a side length of 5 mm. Figure 3 shows the unit cells used in this study.

In the unit cell of the chain, the distance "d" between two links was set to one-third of the link length "L", making up a fixed distance between links. Fig. 3-A shows the distance between blue-green or blue-blue links pairs. This proportion results in a thicker beam, while avoiding neighboring links fusing together. The unit cells were tessellated into a matrix shaped as a rectangular prism (64.10 mm length, 10.13 mm width and 2.60 mm height), as shown in Fig. 3-E. In the tessellation configuration, the unit cell size, space between the unit cells, and lattice thickness can be changed. Table 2 lists the tessellation configurations, dimensions utilized and the volume occupied by the lattice in relation to the volume of the base. The “size in z axis” was defined with the “keep aspect ratio” option turned on, so the entire unit cell was proportionately resized before tessellation. The “spacing” configuration inserts an empty space between the unit cells during the tessellation process, in the (x, y, z) axis, respectively.

**Table 2:** Tessellation design configurations

Unit Cell	Size in z axis (mm)	Spacing (x, y,z) (mm)	Thickness (mm)	% of volume occupied
Chain	1.5	(0, 1, 1)	0.6	12.81%
Knitted	2.0	(0, 0, 1)	0.3	3.19%
Diamond crystal	2.0	(0, 0, 0)	0.3	7.58%
Origami	1.5	(0, 0, 1)	0.75	25.33%

**Fig. 4:**
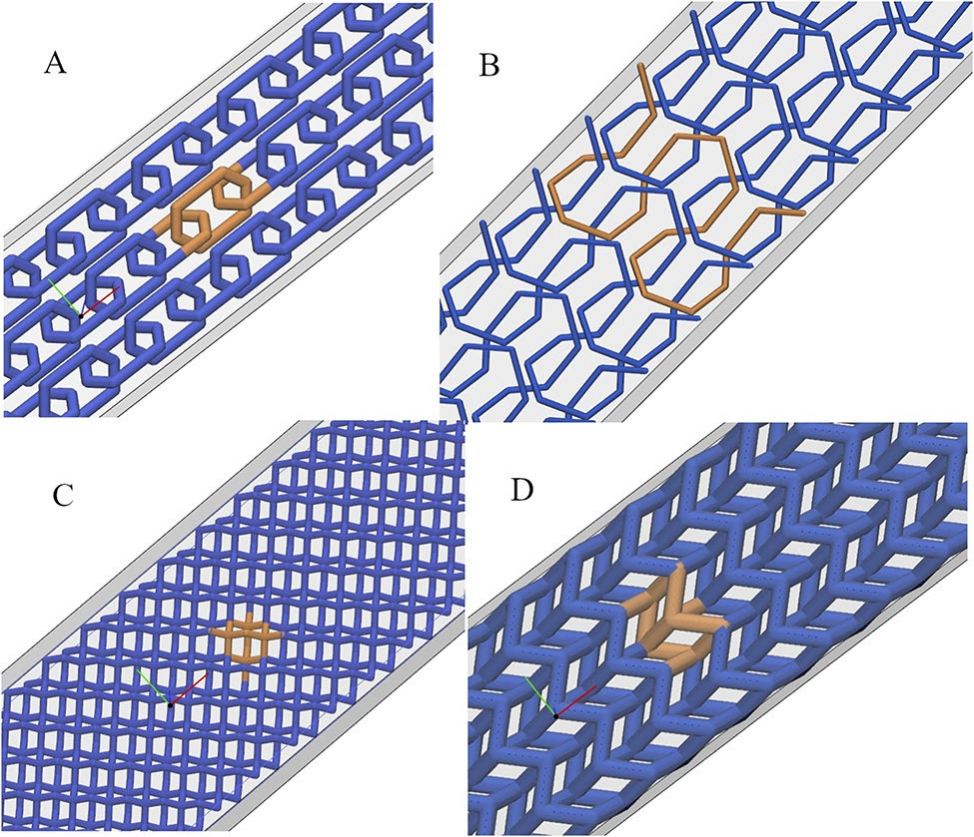
Tesselation designs : (A) chain, (B) knitted pattern, (C) diamond crystal, (D) origami. In (B), one knitted thread is marked. In (A), (C) and (D), one unit cell is marked.

Table 3 lists the printed models, their unit cells, and printing materials of matrix and lattice. Two models were printed with no LR, identified as controls, using only matrix materials. Six samples of each model were printed and tested. Figure 4 shows the tessellation results, with one unit cell marked to depict its repeatability. After printing, the support material was manually removed under running water and tested within 72 h.

**Table 3:** Printed models: unit cells, matrix and lattice materials

Model ID	Unit Cell	Matrix	Lattice
Ctrl_SHA30	Control	SHA30	-
Ch_T600_SHA85	Chain	T600	SHA85
Ch_T600_SDB	Chain	T600	SDB
Knit_SHA30_SHA95	Knitted	SHA30	SHA95
DiaCr_SHA30_SHA70	Diamond crystal	SHA30	SHA70
Ori_SHA30_SHA70	Origami	SHA30	SHA70
Ctrl_T600	Control	T600	-
Knit_T600_SHA85	Knitted	T600	SHA85

### Uniaxial tensile tests of printed materials

Uniaxial tensile tests were performed using Instron® 3365 (Norwood, MA, EUA) and its proprietary software for analysis. Printed samples were held by pneumatic clamps and stretched until rupture at a strain rate of 10 mm/min, according to the ASTM D638 for the specimen type V. The load applied, sample length, stress and strain were recorded.

The thickness of each sample was determined from the average of five measurements taken at the stretching area, that is between the pneumatic clamps used to hold the samples in the equipment. Before testing, the samples were conditioned, simulating testing conditions of biological samples. The conditioning was carried out with a pre-load of 0.02 MPa and three loading-unloading cycles from 0 to 1 N of load at a strain rate of 1 mm/mm/min.

The elastic moduli were determined from the uniaxial tests. The engineering stress ${\sigma }_{E}$ was calculated as the ratio of applied force to initial cross-sectional area, and engineering strain ${\epsilon}_{E}$ was calculated as the ratio of elongation given by the clamp displacement to the initial length [20].

The elastic modulus $E$ is interpreted as the slope from the origin to each point of the stress-strain curve, corresponding to the stiffness of materials with a linear behavior [34]. The incremental elastic modulus was determined for materials with a nonlinear behavior. The incremental elastic modulus ${E}_{inc}$ is the slope of the stress-strain curve at any point, and is calculated as the derivative of the stress-strain curve [35].

**Fig. 5:**
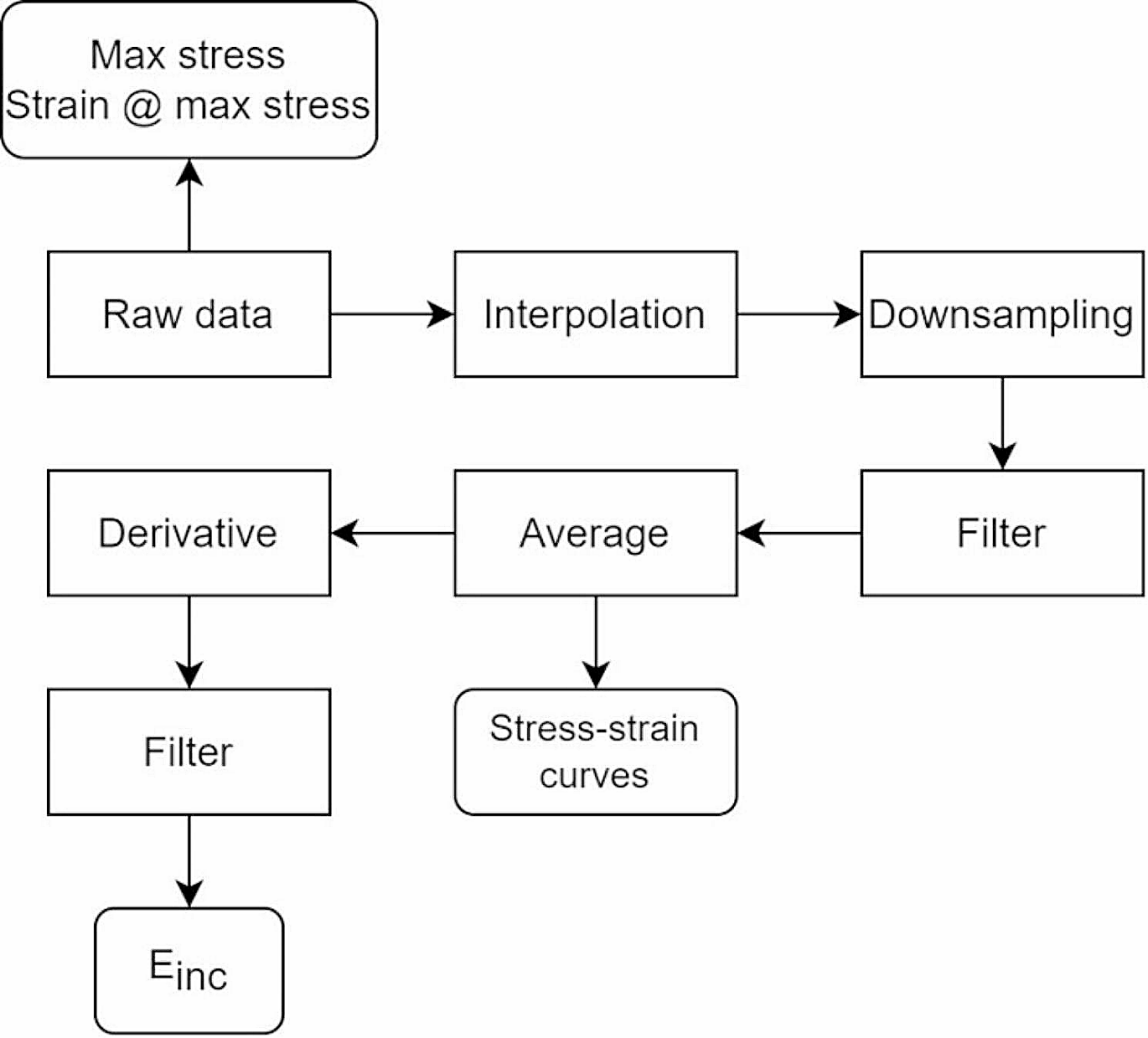
Diagram of process flow used in this study

Fig. 5 shows the flow of steps for analysis of tensile tests (Matlab®; version R2022a). Max stress and strain at max stress values were obtained from the raw data. Stress and strain signals were acquired at a sampling frequency of 100 Hz. Due to the different test durations, interpolation was used to equalize the vector size of all samples. The interpolated data were downsampled by a factor of 10 and filtered (second-order Butterworth low-pass filter, with a 0.1 Hz cutoff frequency). After filtering, the stress and strain signals were averaged and their derivatives were calculated using the central differences and the butterworth filter was reapplied to reduce the noise added by the derivative calculation. The derivatives of stress and strain were used for the calculation of the ${E}_{inc}$.

Statistical analysis was performed with a one-tailed T-test on Matlab® considering a significance of 5%.

## Results and discussion

**Fig. 6:**
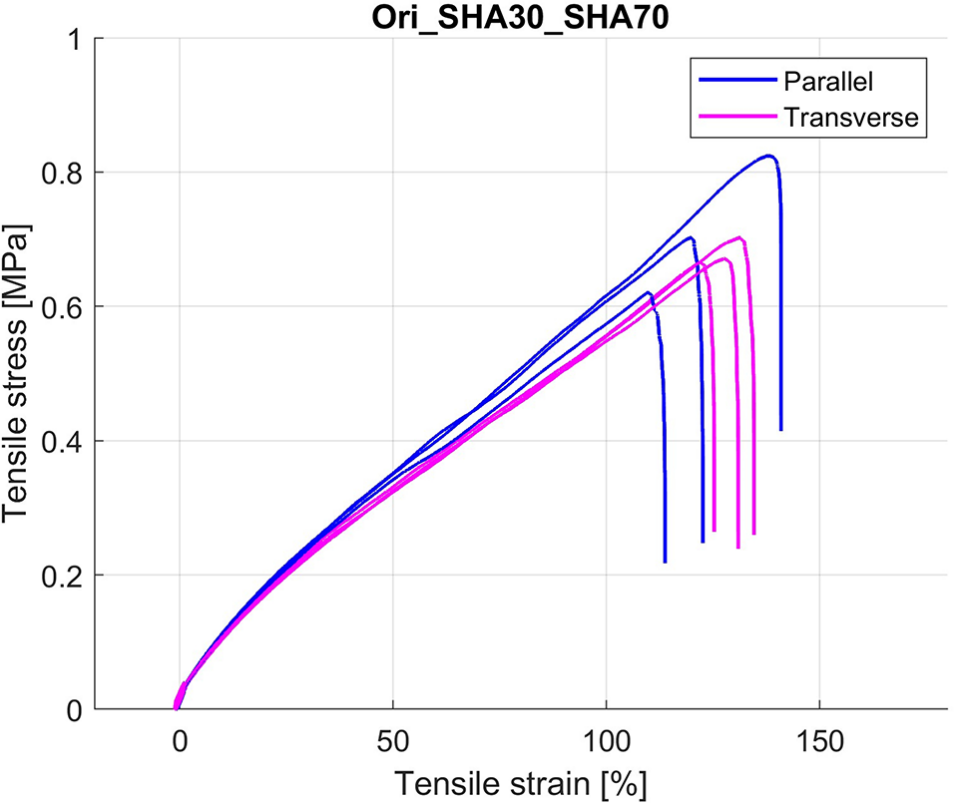
Uniaxial tensile test results for the Ori_SHA30_SHA70 model in different printing orientations

The models were printed with the main axis in the horizontal direction, except for the Ori SHA30_SHA70, printed with the main axis in the vercial direction (transverse). Table 4 shows the maximum stress and strain at maximum stress, and Fig. 6 shows the uniaxial test results as stress-strain graphs in both printing orientations. Previous work [13] shows that for homogenous materials, such as the SHA30, printing directions have limited effect on the maximum stress. It was also demonstrated [14] that considering the interface between a rigid and a flexible material, such as between the matrix and the LR, the printing direction has little influence in the maximum stress.

**Table 4:** Uniaxial tensile test results of the Ori_SHA30_SHA70 model in different printing directions

Print direction	Max stress [MPa]	Strain @ max stress [%]
Parallel	0.72 ± 0.10	122.15 ± 14.19
Transverse	0.68 ± 0.02	126.65 ± 4.67

**Fig. 7:**
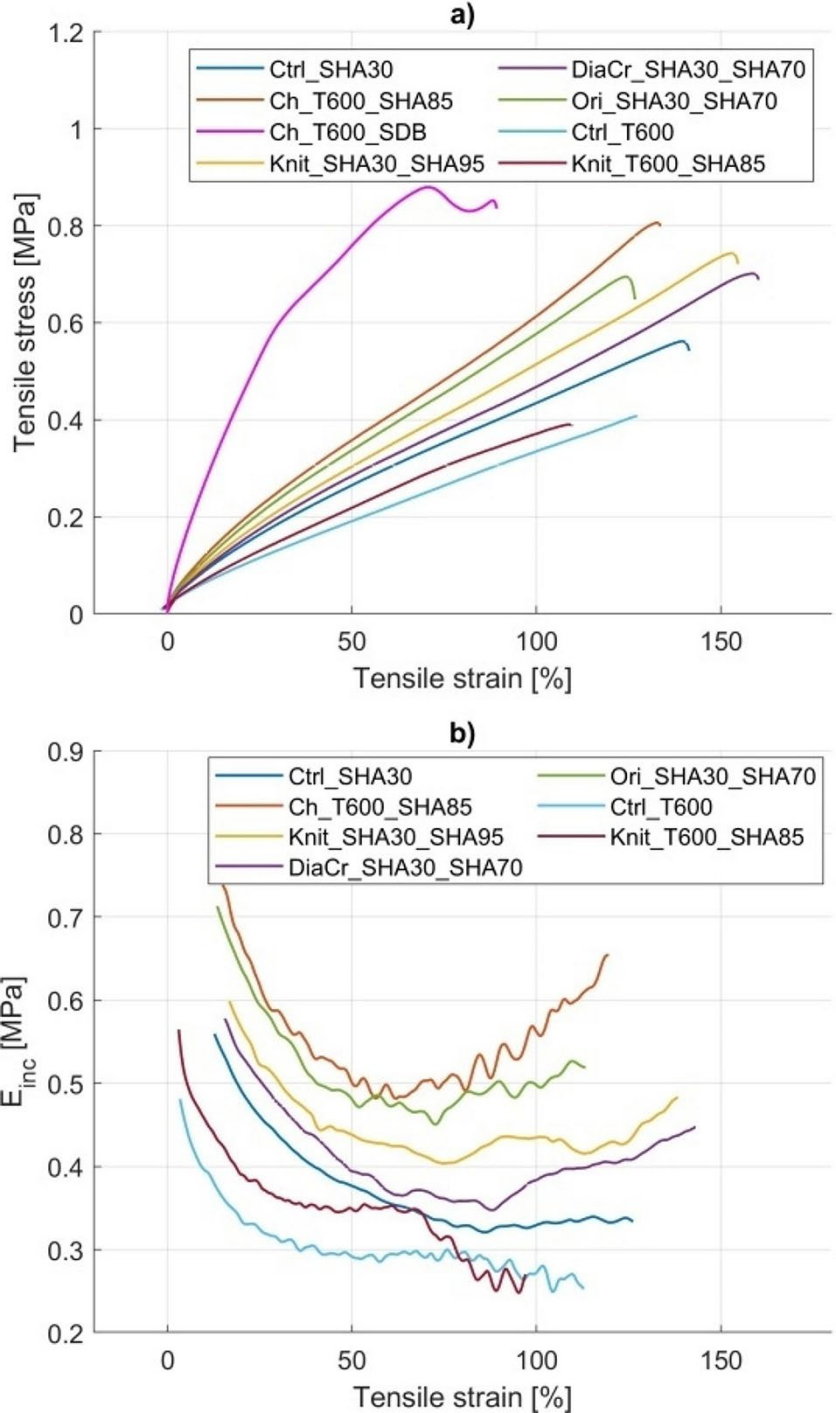
Uniaxial tensile test results. Stress-strain curves (a), ${E}_{inc}$-strain curves (b).

Table 5 lists the maximum stress and strain at the maximum stress for the models. Figure 7 shows the uniaxial tensile tests results as stress-strain and ${E}_{inc}$-strain graphs. Determination of ${E}_{inc}$considered 15 to 90% of the test duration, after conditioning and immediately before the rupture. The behavior of ${E}_{inc}$ of the Ch_T600_SDB model is very similar to the other models tested. The max value of ${E}_{inc}$ of the Ch_T600_SDB is 1.99 MPa at 10.87% strain, and the min ${E}_{inc}$ is -0.75 MPa at 77.06% strain.

**Table 5:** Uniaxial tensile tests results

Model	Max stress [MPa]		Strain @max stress [%]	
	$\mu \pm \sigma$	CoV	$\mu \pm \sigma$	CoV
Ctrl_SHA30	0.56 ± 0.03	0.06	139.46 ± 9.81	0.07
Ch_T600_SHA85	0.81 ± 0.02	0.02	132.95 ± 3.61	0.03
Ch_T600_SDB	0.88 ± 0.02	0.02	70.44 ± 0.86	0.01
Knit_SHA30_SHA95	0.74 ± 0.02	0.03	152.94 ± 5.73	0.04
DiaCr_SHA30_SHA70	0.70 ± 0.05	0.07	158.21 ± 8.99	0.06
Ori_SHA30_SHA70	0.70 ± 0.07	0.10	124.40 ± 9.77	0.08
Ctrl_T600	0.41 ± 0.03	0.07	127.33 ± 7.47	0.06
Knit_T600_SHA85	0.39 ± 0.01	0.01	108.70 ± 5.93	0.05

${\upmu }$ – Mean, ${\upsigma }$ – Standard deviation, CoV – Coefficient of Variation

As seen in Fig. 7, up to approximately 40% strain, models show decreasing ${E}_{inc}$ as strain increases. At strains above 60% initial strain the Ch_T600_SHA85, DiaCr_SHA30_SHA70 and Ori_SHA30_SHA70 models show a slight strain-stiffening behavior, with increasing ${E}_{inc}$ while the Ctrl_SHA30, Ctrl_T600 and Knit_SHA30_SHA95 models have approximately constant ${E}_{inc}$, and the Knit_T600_SHA85 model shows increasing flexibility with decreasing ${E}_{inc}$.

The T600 is formed by a gel-like core surrounded by 600 micrometers thick A30 wall [9]. This wall is printed around all borders of the geometry, internal or external; thus the A30 wall is also printed around the LR of chains and knitting. In the chain design, the A30 wall covers almost the entirety of the printed samples, effectively creating a matrix with A30 only, instead of T600. Therefore, the chain models will be compared with the A30 control model instead of the T600 control model, whereas the Knit_T600_SHA85 model is compared to the Ctrl_T600 model.

The Ctrl_SHA30 model showed greater maximum stress and greater strain at maximum stress than the Ctrl_T600 model, due to its composition.  

Comparing the Ctrl_SHA30 with the Ch_T600_SHA85 and Ch_T600_SDB models, both models with LR have greater maximum stress than controls. Ch_T600_SDB has smaller strain at max stress than the control, but Ch_T600_SHA85 has the same strain at max stress as the control. This indicates that reinforcing the sample with chains increase the mechanical resistance but can reduce the strain at which this max stress is achieved. Earlier studies [13; 36] also showed different LR with a similar behavior, reducing the strain at maximum stress, when compared to samples with no reinforcement lattice.

Comparing the Ch_T600_SHA85 and Ch_T600_SDB models, the Ch_T600_SDB shows greater maximum stress, but smaller strain at maximum stress. This indicates that the material used as LR affects the mechanical resistance and a stiffer LR results in a stiffer model.

Comparing the Ctrl_SHA30 with the Ch_T600_SHA85, the Knit_SHA30_SHA95, DiaCr_SHA30_SHA70 and Ori_SHA30_SHA70 models, the models with LR showed greater maximum stress compared to the control model. For strain at maximum stress, the Ori_SHA30_SHA70 model is smaller than the control model, whereas the DiaCr_SHA30_SHA70 and the Knit_SHA30_SHA95 models are greater. The Ch_T600_SHA85 model exhibited the same strain at max stress. It should be noted that strain at max stress is inversely proportional to the thickness of the LR and the percentage of the matrix volume occupied by the LR, indicating that the amount of rigid LR inside a flexible matrix affects the strain achieved at max stress. Previous studies [13] showed a similar result for a sample with rigid ellipsoidal particles immersed in a flexible matrix compared to a similar sample without the particles, and that increasing thickness increases the rigidity of the printed sample [17].

Comparing the Ctrl_SHA30 and Knit_SHA30_SHA95 pair with Ctrl_T600 and Knit_T600_SHA85 pair, the model with LR from the A30 matrix pair has greater maximum stress and greater strain at maximum stress than its control model, whereas, in the T600 matrix pair, the model with LR has equal maximum stress and smaller strain at maximum stress compared to the control model. This indicates that the effect of the LR depends on the matrix material.

In summary, all printed models show similar reduction in ${E}_{inc}$for strains up to approximately 50%; LR increased rigidity in all models resulting in greater ${E}_{inc}$ compared to their controls printed with the same matrix, up to 50% strain; LR can increase max stress. In contrast, strain at maximum stress depends on the design and material of the LR and also seems to be affected by the matrix material.

**Fig. 8:**
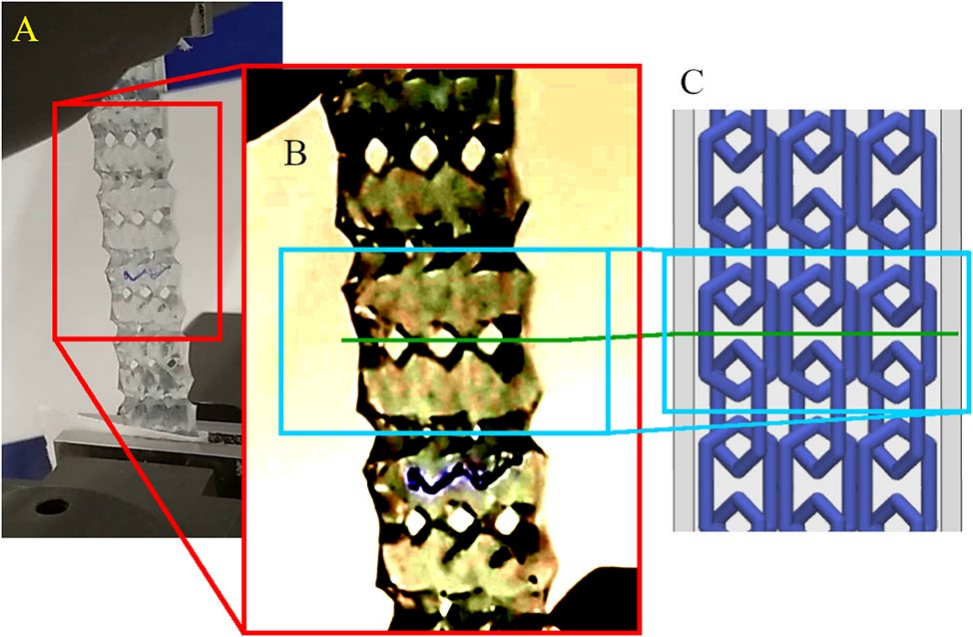
Detail of the Ch_T600_SDB model during tensile test showing the moment the matrix ruptured but not the LR (A), a top view of the lattice magnified (B), and the site of rupture indicated by a green line on top of the design of the lattice (C)

The behavior of the Ch_T600_SDB model stands out. Around 70% strain, the stress of the model starts dropping up to 80% strain, reflected as the negative ${E}_{inc}$ value mentioned before. At this strain range, most of the matrix of the samples ruptured, while the LR remained connected. This indicates that when the LR material is much more rigid than the matrix material, the matrix can rupture before the LR. The final stretch, above 90% strain until the LR finally ruptured, is most likely caused by the stretch of the LR. Figure 8-A shows one sample of the model during the tensile test when the base material ruptured but the LR did not. The matrix and the LR have similar colors, so the LR is barely visible during the tensile test and the Fig. 8-B was edited to increase the LR visibility. In Fig. 8-C, the 3D model of the lattice is seen and the green line indicates where the matrix ruptured.

**Fig. 9:**
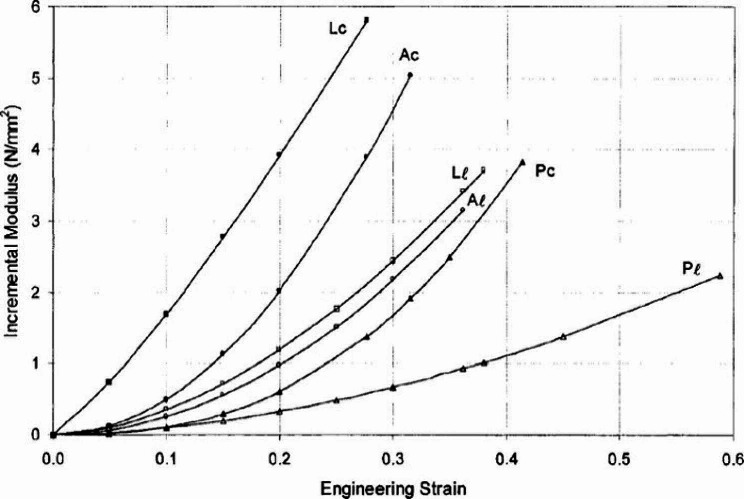
Incremental modulus (${E}_{inc}$) versus strain, in different regions. L, A, P represent, respectively, lateral, anterior and posterior regions, while subscripts c and l represent, respectively, circumferential and longitudinal directions. Reproduced from Thubrikar et al. (2001) [35] with permission from Taylor & Francis

Thubrikar et al. (2001) [35] performed uniaxial tensile tests on abdominal aortic aneurysm (AAA) samples from patients undergoing elective surgical repair. These samples were extracted in rectangular shapes from different regions of the abdominal aorta in both longitudinal and circumferential orientations. Figure 9 shows the average ${E}_{inc}$-strain of an abdominal aortic aneurysm, in different regions and directions. The effect of the strain-stiffening behavior of soft tissue on the ${E}_{inc}$ is shown in the curves in Fig. 9, with lower values at smaller strains, and their increase as the strain increases.

Although Thubrikar et al. (2001) [35] only studied AAAs, Fig. 9 illustrates the similarities between an aneurysmal and normal aorta. Therefore, it is expected that a model of a normal aorta behaves similarly to the curves in Fig. 9, but with a lower slope.

As seen in Fig. 7-B, the printed models studied did not reproduce the strain-stiffening behavior exhibited by the biological aortic tissue, shown in Fig. 9.

Table 6 shows the maximum stress and strain at maximum stress values, from normal and aneurysmal aortas (both abdominal and thoracic), in longitudinal and circumferential orientations [37–41].

**Table 6:** Uniaxial tensile test results for healthy and aneurysmal aortas, from the references

Reference	Healthy / Aneurysm	Orientation	Max Stress [MPa]		Strain @ max stress [%]	
			$\mu \pm \sigma$	CoV	$\mu \pm \sigma$	CoV
Forsell et al. (2014) * [37]	Ascending TAA	Circ.	0.486 ± 0.21	0.43	52.11 ± 0.13 **	0.002
Forsell et al. (2012) [38]	AAA	Long.	0.437 ± 0.319	0.73	32.5 ± 11.3 **	0.35
Maizato et al. (2023) *** [39]	HT	Circ.	0.76 ± 0.23	0.30	57.18 ± 7.96	0.14
Reeps et al. (2012) [40]	AAA	Circ.	1.063 ± 0.49	0.46	-	-
Vallabhaneni et al. (2004) [41]	AAA	Long.	0.53 ± 0.02	0.04	30 ± 2	0.07
	HA	Circ.	0.61 ± 0.07	0.11	29 ± 4	0.14
	HA	Long.	1.30 ± 0.11	0.08	33 ± 4	0.12

$\mu$ – Mean, $\sigma$ – Standard deviation, CoV – Coefficient of Variation, TAA – Thoracic Aortic Aneurysm, AAA – Abdominal Aortic Aneurysm, HA – Healthy Abdominal aorta, HT – Healthy Thoracic aorta, Circ – Circumferential, Long – Longitudinal. * Data retrieved from supplementary material document. ** Data given as relative stretch, adapted to strain. *** Mean from two aortas, with three samples each

Comparing the maximum stress of the printed models with the references from Table 6:

- The Ctrl_SHA30, Ctrl_T600 and Knit_T600_SHA85 models were similar to those of the ascending thoracic aortic aneurysm reported by Forsell et al. (2014) [37];
- The Ctrl_SHA30, Ctrl_T600 and Knit_T600_SHA85 models were equal to the AAA in the axial direction reported by Forsell et al. (2012) [38];
- The Ch_T600_SHA85, Ch_T600_SDB, Knit_SHA30_SHA95, DiaCr_SHA30_SHA70 and Ori_SHA30_SHA70 models are equal to the healthy thoracic aorta in the circumferential direction in Maizato et al. (2023) [39];
- The Ch_T600_SHA85, Ch_T600_SDB and Knit_SHA30_SHA95 models are equal to the AAA in the circumferential direction reported by Reeps et al. (2012) [40];
- The Ctrl_SHA30 model is equivalent to the healthy abdominal aorta in the circumferential direction from Vallabhaneni et al. (2004) [41].

The printed models showed greater strain at maximum stress than materials listed in the references of Table 6.

In general, the printed models in Table 5 also show smaller coefficient of variation for maximum stress and strain at maximum stress compared to the references in Table 6, indicating that the printed models are more consistent for testing.

**Fig. 10:**
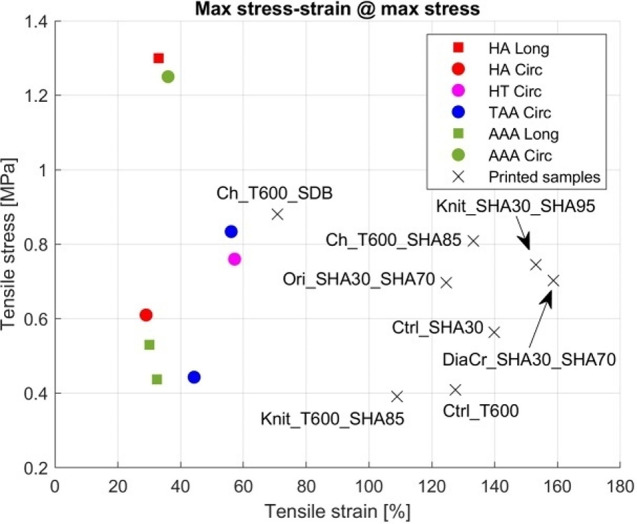
Stress-strain values at max stress reported in the literature and of the printed models. TAA – Thoracic Aortic Aneurysm, AAA – Abdominal Aortic Aneurysm., HA – Healthy Abdominal aorta, HT – Healthy Thoracic aorta, Circ – Circumferential, Long – Longitudinal

Table 5 summarizes the comparison of stress obtained in printed models with data from the literature. Fig. 10 shows a comparison of the maximum stress and strain at maximum stress of the printed models listed in Table 6 and the biological tissues listed in Table 7. It is seen that maximum stresses are similar but the strain at maximum stress of the printed models is larger than that of biological tissues.?

**Table 7:** Printed models closest to the references for max stress

Reference	Healthy / Aneurysm	Orientation	Models equal in max stress
Forsell et al. (2014) [37]	Ascending TAA	Circ.	Ctrl_SHA30, Ctrl_T600 and Knit_T600_SHA85
Forsell et al. (2012) [38]	AAA	Long.	Ctrl_SHA30, Ctrl_T600 and Knit_T600_SHA85
Maizato et al. (2023) [39]	HT	Circ.	Ch_T600_SHA85, Ch_T600_SDB, Knit_SHA30_SHA95, DiaCr_SHA30_SHA70 and Ori_SHA30_SHA70
Reeps et al. (2012) [40]	AAA	Circ.	Ch_T600_SHA85, Ch_T600_SDB and Knit_SHA30_SHA95
Vallabhaneni et al. (2004) [41]	AAA	Long.	Ctrl_SHA30*
	HA	Circ.	Ctrl_SHA30
	HA	Long.	Ch_T600_SDB*

TAA – Thoracic Aortic Aneurysm, AAA – Abdominal Aortic Aneurysm., HA – Healthy Abdominal aorta, HT – Healthy Thoracic aorta, Circ – Circumferential, Long – Longitudinal. *No printed model is equal, closest model

Figure 10 shows a comparison of the maximum stress and strain at maximum stress of the printed models shown in Table 5 and the biological tissue from Table 6. Although the maximum stresses are at similar levels, the strain at maximum stress of the printed models is larger than the biological tissues.

### Aortic wall model using metamaterials

**Fig. 11:**
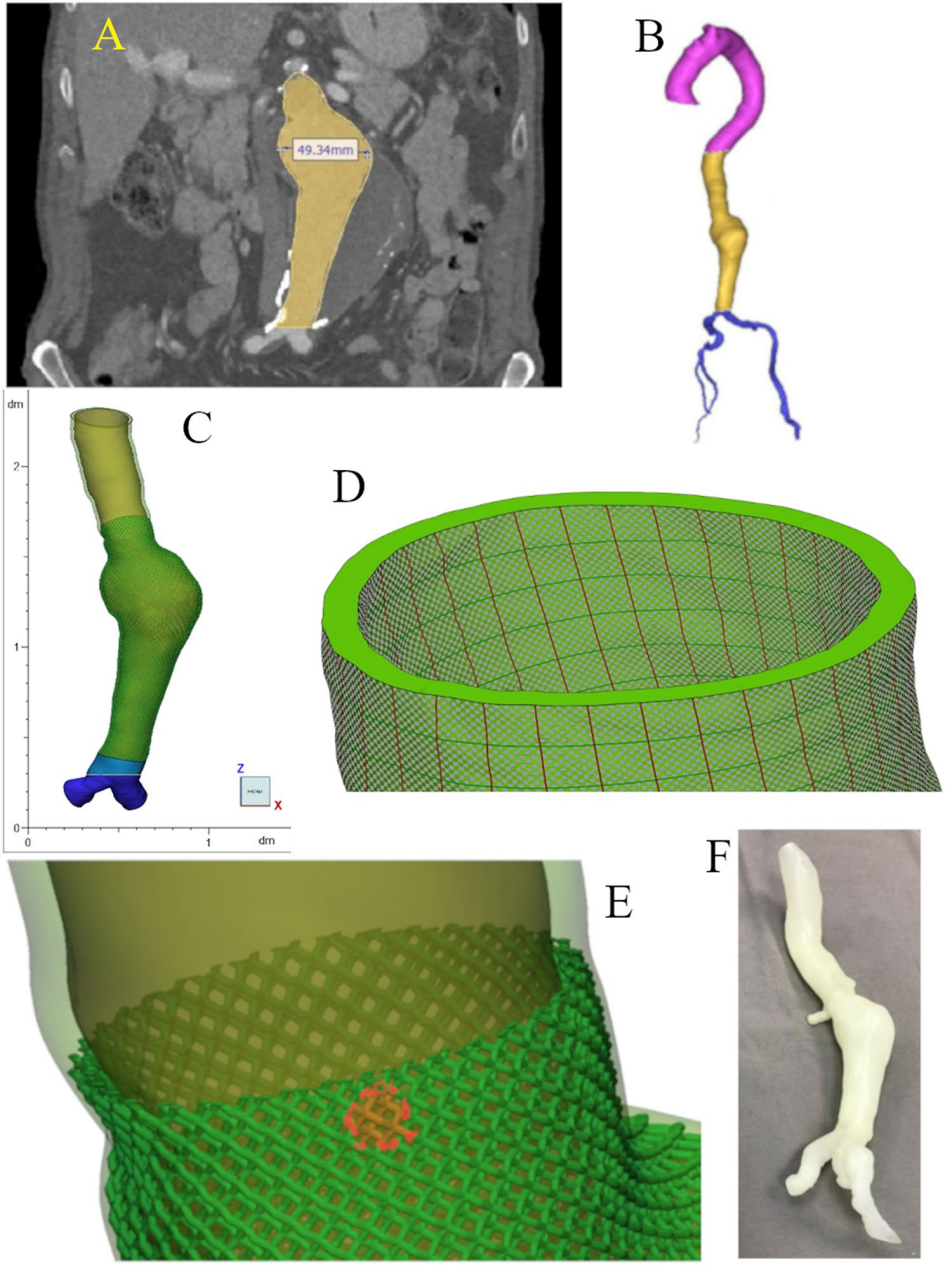
(A) Coronal view of the CT image showing the region of the aorta (in yellow) and the maximum diameter of the aneurysm. (B) 3D model of the segmentation, divided in the ascendant and the thoracic aorta (pink), abdominal aorta (yellow), and iliac arteries (blue) sections. (C) Final model with the LR in the aortic wall. (D) UV maps created to align the unit cell to the vessel wall. (E) Detail of the final model with and without the LR, including one marked unit cell. (F) Photograph of the printed model of the abdominal aortic aneurysm withn no reinforcement.

The angiographic CT scan image of an anonimized patient with abdominal aneurysm was used under the guidelines of the local ethics committee. The image from the CT scan was composed of 2213 slices of 1 mm thickness, 512 × 512 pixels each slice, each pixel corresponding to 0.97632 mm. After segmentation, the model was exported to a CAD software to insert the metamaterial into the aortic wall. Figure 11-F shows the image of the aortic vessel wall printed with A30 and no LR. For image processing and lattice design the commercial software Mimics® (version 25.0) [42] and 3-matic® were used, respectively.

The “segment vessel” function, that semi-automatically segments a region based on the Hounsfield Units (HU) intensity, an initial point, and a direction, was used. First, the “Aorta – Main Branch” configuration was used considering the initial point close to the aortic root, directed towards the aortic arch and HU values between 230 and 500. This first portion corresponds to the aorta from the aortic root to one of the iliac arteries. The same function was utilized with a “Renal artery” configuration and the start of the unsegmented iliac artery was selected as the initial point, including the second iliac artery. Oversegmented regions were manually removed. Finally, the segmentation was divided in two regions at the diaphragm level and 5 mm above the bifurcation of the aorta into the iliac arteries, dividing the segmentation in thoracic aorta, abdominal aorta and the iliac arteries.

To simplify the metamaterial insertion process in the wall, only part of the abdominal aorta and the iliac arteries were used. The iliac arteries were cut at 15 mm below the bifurcation of the aorta.

Three planes perpendicular to the centerline of the segmentation were created: at the thoracic aorta, iliac arteries and above the aneurysm. The two first planes were created to trim the aorta, reducing the working area, and to help align the UV maps, and the last plane to limit the aneurysm portion.

The segmentation was performed on an angiographic CT and it created a model of the blood pool inside the vessel, not a model of the vessel wall. To create the vessel wall a 2 mm offset of the blood pool was applied to the blood pool model.

For simplicity, the unit cell chosen for this model was the diamond crystal, owing to its symmetry. To tesselate the unit cell in the vessel wall created, UV maps on the non-planar surfaces were generated, which were used to align the unit cell to the surface of the wall, and were aligned with the planes previously created and to a projection of the centerline on the vessel wall. For the inner side of the wall, both the UV map “XYZ to U factor” and “XYZ to V factor” were defined as 2.75 mm, and the resulting “Size U”, “Size V”, “Position U” and “Position V” of the internal map after automatic alignment were copied to the map on the outside of the vessel wall. Then, the maps were optimized with “angles and area” for 100 iterations, and a final alignment is shown in Fig. 11-D. The command “UV Based Conformal Lattice” tesselated the diamond crystal unit cell on the UV maps, with a height offset of 0.5, and untrimmed “Desired result”. Then, using the plane above the aneurysmatic portion, the resulting lattice was reduced to the aneurysm and below. The thickness of the lattice was defined as 0.5 mm and inserted in the aortic vesse wall.

During the process described above, a part of the aorta was trimmed off, creating an space between the aneurysm and the iliac arteries with no vessel wall, so the “loft” command was used to close this gap. The “loft” was used with the “smooth” method, in the “orthogonal” direction, and the adequate curves as the references. As shown in Fig. 11-C, the final result includes the aortic vessel wall, the LR, the iliac arteries wall, and the loft region, ready to be exported as a STL file to GrabCAD® and printed. It is important to note that the segmented blood pool must also be included, so that an easy-to-remove material can be selected for the internal filling. Figure 11-E shows a detailed view of the model with and without the LR.

## Conclusions

This work describes the application of the 3D printing technology to model the aorta.

Experiments were conducted using four unit cells: chain [32], knitted pattern [26], origami pattern known as Miura-ori [33], and a diamond crystal. The chain, knitted pattern and the origami designs were customized, while the diamond crystal was loaded from 3-matic®’s [31] library. The samples were printed with Stratasys® J750™ DAP [29] using VCyan, VMgnt, A30, TMat, GMat and BMat materials [28], combined to achieve various hardness values.

The stress-strain behavior of the printed samples was determined using uniaxial tensile tests, and the ${E}_{inc}$ was calculated. The results of the models were compared in groups that had the same matrix material but different LR designs, or models with the same matrix material and LR design but different LR material. All models exhibited similar reduction in at strains lower than 40%; the use of LR increases rigidity, with models with LR showing greater compared to the non-reinforced model of the same matrix, up to 50% strain. LR can increase maximum stress, but strain at maximum stress could increase or decrease, depending on the LR volume, design, material; and the effect of LR on mechanical behavior also seems to depend on the matrix material. Printed models showed maximum stress ranging from 0.39 ± 0.01 MPa to 0.88 ± 0.02 MPa, and strain at maximum stress ranging from 70.44 ± 0.86% to 158.21 ± 8.99%.

The chain and knitted models rely primarily on the relative movement between their links or threads. In this study, the chain and knitted pattern were utilized as LR within the solid matrix, so the movement of their links/threads was limited by the stretching of the matrix. Similarly, the origami pattern [33] and diamond crystal primarily depend on the deformation of their structure, which is also limited by the stretching of the matrix. A more detailed analysis of the mechanical behavior using computational simulations and finite element methods [43] may provide further insight.

The results from previous studies using samples of healthy and aneurysmal aortas extracted from the thoracic and abdominal regions in the longitudinal and circumferential directions [37; 38; 39; 40; 41] were compared to the printed models. The printed models displayed maximum stress comparable to at least one of the biological tissues in the studies cited in the references, despite having different strains at maximum stress. More importantly, the printed samples did not exhibit strain-stiffening behavior. Previous studies show the effects of reinforcement on the mechanical properties of 3D printed samples with ellipsoidal particles aligned parallel to the tensile stress direction [13], and Peano curves to design the reinforcement [36]. Wang, Zhao, et al. (2016) [17] and Wang, Wu, et al. (2016) [18] studied 3D printed sinusoidal wave and helical helix reinforcement designs to mimic soft tissues, and Chen et al. (2018) [43] studied a statistical approach of these reinforcements to optimize their designs to mimic the stress-strain of a desired tissue.

The use of a rigid polymer printed as LR inside a more flexible one was investigated. The LR was designed in a way that its structure affects its mechanical properties, known as a metamaterial [17, 18]. Zhalmuratova et al. (2019) [26] proposed a reinforcing approach with elastomeric materials enveloping commercial fabrics, which was compared with the biological aortic tissue.

A simplified virtual model of the aorta with abdominal aneurysm, in which a lattice was included in the vessel wall as LR, was created. A symmetric unit cell was chosen to simplify the insertion process, which is long and prone to errors. For an assymetric unit, the UV map should be positioned carefully to properly align the LR. The tesselation of the unit cell on areas with larger curvature may result in errors, such as intersecting triangles.

The mechanical behavior of the printed models did not perfectly replicate the aortic vessel wall, however, incorporating LR can potentially lead to the development of an aorta model with distinct behavior in each segment.

## Data Availability

Results of uniaxial tensile tests and metamaterial designs are available under request to the corresponding author.

## Abbreviations

3D: Three-dimensional
CT: Computed tomography
DAP: Digital Anatomy™ Printer
LR: Lattice-reinforcement
VCyan: VeroCyan™
VMgnt: VeroMagenta™
A30: Agilus30™
TMat: TissueMatrix™
GMat: GelMatrix™
BMat: BoneMatrix™
SHAXX: SH-AXX (XX represents Shore hardness)
T600: Tissue 600
SDB: Slightly dense bone
*σ*_E_: Engineering stress
∈_E_: Engineering strain
*E*: Elastic modulus
*E*_inc_: Incremental Elastic modulus
CAD: Computer Aided Design
HT: Healthy Thoracic aorta
TAA: Thoracic Aortic Aneurysm
AAA: Abdominal Aortic Aneurysm
HA: Healthy Abdominal aorta
Circ: Circumferential
Long: Longitudinal
HU: Hounsfield Units

## References

[1] Smith CF, Tollemache N, Covill D, Johnston M. Take away body parts! An investigation into the use of 3D-printed anatomical models in undergraduate anatomy education. Anat Sci Educ. 2017;11:44–53. 28753247 10.1002/ase.1718

[2] Fischer S, Romano G, Sharan L, Warnecke G, Mereles D, Kretzler M, et al. Surgical Rehearsal for mitral valve repair: personalizing Surgical Simulation by 3D Printing. Ann Thorac Surg. 2023;115:1062–7. 36638948 10.1016/j.athoracsur.2022.12.039

[3] Torres I, De Luccia N. Artificial vascular models for endovascular training (3D printing). Innovative Surgical Sciences [Internet]. 2018;3:225–34. https://www.degruyter.com/document/doi/10.1515/iss-2018-0020/html. 10.1515/iss-2018-0020 PMC6604582 31579786

[4] Biglino G, Verschueren P, Zegels R, Taylor AM, Schievano S. Rapid prototyping compliant arterial phantoms for in-vitro studies and device testing. J Cardiovasc Magn Reson. 2013;15. 10.1186/1532-429X-15-2 PMC3564729 23324211

[5] Vukicevic M, Mosadegh B, Min JK, Little SH. Cardiac 3D Printing and its Future Directions. JACC: Cardiovascular Imaging [Internet]. 2017;10:171–84. http://imaging.onlinejacc.org/content/10/2/171.full. 10.1016/j.jcmg.2016.12.001 PMC5664227 28183437

[6] Yildiz O, Kose B, Tanidir IC, Pekkan K, Guzeltas A, Haydin S. Single-center experience with routine clinical use of 3D technologies in surgical planning for pediatric patients with complex congenital heart disease. Diagnostic and Interventional Radiology [Internet]. 2021;27:488–96. https://www.dirjournal.org/en/single-center-experience-with-routine-clinical-use-of-3d-technologies-in-surgical-planning-for-pediatric-patients-with-complex-congenital-heart-disease-132440. 10.5152/dir.2021.20163 PMC8289433 34313233

[7] Tong Y-H, Yu T, Zhou M-J, Liu C, Zhou M, Jiang Q et al. Use of 3D Printing to Guide Creation of Fenestrations in Physician-Modified Stent-Grafts for Treatment of Thoracoabdominal Aortic Disease. Journal of Endovascular Therapy: An Official Journal of the International Society of Endovascular Specialists [Internet]. 2020 [cited 2022 Apr 6];27:385–93. https://pubmed.ncbi.nlm.nih.gov/32517556/. 10.1177/1526602820917960 32517556

[8] Bezek LB, Cauchi MP, De Vita R, Foerst JR, Williams CB. 3D printing tissue-mimicking materials for realistic transseptal puncture models. J Mech Behav Biomed Mater. 2020;110:103971. 32763836 10.1016/j.jmbbm.2020.103971

[9] Severseike L, Lee V, Brandon T, Bakken C, Bhatia V. Polyjet 3D printing of tissue-mimicking materials: how well can 3D printed synthetic myocardium replicate mechanical properties of organic myocardium? bioRxiv. 2019.

[10] Maragiannis D, Jackson MS, Igo SR, Chang SM, Zoghbi WA, Little SH. Functional 3D printed patient-specific modeling of severe aortic stenosis. J Am Coll Cardiol. 2014;64:1066–8. 25190245 10.1016/j.jacc.2014.05.058

[11] Lee J-Y, An J, Chua CK. Fundamentals and applications of 3D printing for novel materials. Appl Mater Today. 2017;7:120–33. 10.1016/j.apmt.2017.02.004

[12] Kaschwich M, Horn M, Matthiensen S, Stahlberg E, Behrendt C-A, Matysiak F, et al. Accuracy evaluation of patient-specific 3D-printed aortic anatomy. Annals Anat - Anatomischer Anzeiger. 2021;234:151629. 10.1016/j.aanat.2020.151629 33137459

[13] Tee YL, Peng C, Pille P, Leary M, Tran P. PolyJet 3D Printing of Composite materials: experimental and Modelling Approach. JOM. 2020;72:1105–17. 10.1007/s11837-020-04014-w

[14] Lumpe TS, Mueller J, Shea K. Tensile properties of multi-material interfaces in 3D printed parts. Mater Design. 2019;162:1–9. 10.1016/j.matdes.2018.11.024

[15] Kwon J, Ock J, Kim N. Mimicking the Mechanical Properties of Aortic Tissue with pattern-embedded 3D Printing for a realistic Phantom. Materials. 2020;13:5042. 33182404 10.3390/ma13215042 PMC7664885

[16] Cloonan AJ, Shahmirzadi D, Li RX, Doyle BJ, Konofagou EE, McGloughlin TM. 3D-Printed tissue-mimicking phantoms for medical imaging and computational validation applications. 3D Print Additive Manuf. 2014;1:14–23. 10.1089/3dp.2013.0010 PMC4981152 28804733

[17] Wang K, Zhao Y, Chang Y-H, Qian Z, Zhang C, Wang B et al. Controlling the mechanical behavior of dual-material 3D printed meta-materials for patient-specific tissue-mimicking phantoms. Materials & Design [Internet]. 2016 [cited 2019 Dec 31];90:704–12. https://www.sciencedirect.com/science/article/abs/pii/S0264127515307644.

[18] Wang K, Wu C, Qian Z, Zhang C, Wang B, Vannan MA. Dual-material 3D printed metamaterials with tunable mechanical properties for patient-specific tissue-mimicking phantoms. Additive Manufacturing [Internet]. 2016 [cited 2019 Jul 29];12:31–7. https://www.sciencedirect.com/science/article/pii/S221486041630118X.

[19] Zhalmuratova D. Reinforced Elastomer Composites and Metamaterials for Neo-aorta Applications [Internet] [Thesis]. [University of Alberta]; 2019. https://era.library.ualberta.ca/items/ca57f124-57fa-4472-88e5-a97e4b345260.

[20] Wang X, Carpenter HJ, Ghayesh MH, Andrei Kotousov, Zander AC, Amabili M, et al. A review on the biomechanical behaviour of the aorta. J Mech Behav Biomed Mater. 2023;144:105922–2. 37320894 10.1016/j.jmbbm.2023.105922

[21] Bossone E, Eagle KA. Epidemiology and management of aortic disease: aortic aneurysms and acute aortic syndromes. Nature Reviews Cardiology [Internet]. 2020;18:1–18. https://www.nature.com/articles/s41569-020-00472-6. 10.1038/s41569-020-00472-6 33353985

[22] JCS Joint Working Group. Guidelines for Diagnosis and Treatment of Aortic Aneurysm and Aortic Dissection (JCS 2011). Circulation Journal [Internet]. 2013;77:789–828. https://www.jstage.jst.go.jp/article/circj/77/3/77_CJ-66-0057/_pdf. 10.1253/circj.cj-66-0057 23412710

[23] Wang Z, You Y, Yin Z, Bao Q, Lei S, Yu J et al. Burden of aortic aneurysm and its attributable risk factors from 1990 to 2019: an analysis of the global burden of Disease Study 2019. Front Cardiovasc Med. 2022;9. 10.3389/fcvm.2022.901225 PMC9197430 35711350

[24] He CM, Roach MR. The composition and mechanical properties of abdominal aortic aneurysms. Journal of Vascular Surgery [Internet]. 1994 [cited 2021 Jun 27];20:6–13. https://www.jvascsurg.org/article/0741-5214(94)90169-4/fulltext. 10.1016/0741-5214(94)90169-4 8028090

[25] Holzapfel GA, Gasser TC, Ogden RW. A New Constitutive Framework for Arterial Wall Mechanics and a Comparative Study of Material Models. Journal of elasticity and the physical science of solids [Internet]. 2000;61:1–48. 10.1023/A:1010835316564.

[26] Zhalmuratova D, La T-G, Yu KT-T, Szojka ARA, Andrews SHJ, Adesida AB, et al. Mimicking J-Shaped and anisotropic stress–strain behavior of Human and Porcine Aorta by Fabric-Reinforced Elastomer composites. ACS Appl Mater Interfaces. 2019;11:33323–35. 31464413 10.1021/acsami.9b10524

[27] Aremu AO, Brennan-Craddock JPJ, Panesar A, Ashcroft IA, Hague RJM, Wildman RD et al. A voxel-based Method of Constructing and Skinning Conformal and Functionally Graded Lattice Structures Suitable for Additive Manufacturing. Additive Manufacturing [Internet]. 2017;13:1–13. https://www.sciencedirect.com/science/article/pii/S2214860416302810.

[28] Stratasys Ltd. PolyJet Materials | StratasysTM Support Center [Internet]. Stratasys. [cited 2023 Dec 20]. https://support.stratasys.com/en/Materials/PolyJet.

[29] Stratasys Ltd. J750 Digital Anatomy [Internet]. Stratasys. [cited 2022 Dec 15]. https://support.stratasys.com/en/printers/polyjet-legacy/j750-digital-anatomy.

[30] GrabCAD. GrabCAD Print [Internet]. grabcad.com. Stratasys Inc.; [cited 2024 Jan 7]. https://grabcad.com/print.

[31] Materialise. Materialise 3-matic [Internet]. Materialise 3-matic. [cited 2024 Jan 13]. https://www.materialise.com/en/industrial/software/3-matic.

[32] Silveira JFRV, Muniz AR. Chain- and chainmail-like Nanostructures from Carbon Nanotube Rings. Computational Materials Science [Internet]. 2019;161:76–82. https://www.sciencedirect.com/science/article/pii/S0927025619300564.

[33] Islam M, Flach J, Martinez-Duarte R. Carbon origami: a method to fabricate lightweight carbon cellular materials. Carbon. 2018;133:140–9. 10.1016/j.carbon.2018.03.033

[34] Chirinos JA. Arterial stiffness: basic concepts and measurement techniques. Journal of Cardiovascular Translational Research [Internet]. 2012 [cited 2023 Jan 20];5:243–55. https://pubmed.ncbi.nlm.nih.gov/22447229. 10.1007/s12265-012-9359-6 22447229

[35] Thubrikar MJ, Labrosse M, Robicsek F, Al-Soudi J, Fowler B. Mechanical properties of Abdominal aortic aneurysm wall. J Med Eng Technol. 2001;25:133–42. 11601439 10.1080/03091900110057806

[36] Wu C, Do TT, Tran P. Mechanical properties of PolyJet 3D-Printed composites inspired by space-filling Peano Curves. Polymers. 2021;13:3516. 34685275 10.3390/polym13203516 PMC8538836

[37] Forsell C, Björck HM, Eriksson P, Franco-Cereceda A, Gasser TC. Biomechanical properties of the thoracic Aneurysmal Wall: differences between bicuspid aortic valve and tricuspid aortic valve patients. Ann Thorac Surg. 2014;98:65–71. 24881863 10.1016/j.athoracsur.2014.04.042

[38] Forsell C, Swedenborg J, Roy J, Gasser TC. The quasi-static failure properties of the abdominal aortic aneurysm Wall estimated by a mixed Experimental-Numerical Approach. Ann Biomed Eng. 2012;41:1554–66. 23263935 10.1007/s10439-012-0711-4

[39] Maizato MJS, Gutierrez PS, Bacht S, Cestari IN, Sales E, Mazzetto M et al. Towards the Development of 3DPrinted Model of the Human Aorta: Comparison of Mechanical Properties of Biological and Polymeric Materials. In: Luiz J, Rodrigues CR, Ota D, Neto M, Ojeda G, editors. IX Latin American Congress on Biomedical Engineering and XXVIII Brazilian Congress on Biomedical Engineering [Internet]. Cham: Springer Nature Switzerland; 2024 [cited 2024 Jan 9]. pp. 388–95. 10.1007/978-3-031-49401-7_39.

[40] Reeps C, Maier A, Pelisek J, Härtl F, Grabher-Meier V, Wall WA, et al. Measuring and modeling patient-specific distributions of material properties in abdominal aortic aneurysm wall. Biomech Model Mechanobiol. 2012;12:717–33. 22955570 10.1007/s10237-012-0436-1

[41] Vallabhaneni SR, Gilling-Smith GL, How TV, Carter SD, Brennan JA, Harris PL. Heterogeneity of Tensile Strength and Matrix Metalloproteinase Activity in the Wall of Abdominal aortic aneurysms. J Endovasc Ther. 2004;11:494–502. 15298501 10.1583/04-1239.1

[42] Mimics. Materialise Mimics | 3D Medical Image Processing Software [Internet]. Materialise Mimics. [cited 2024 Jan 13]. https://www.materialise.com/en/healthcare/mimics-innovation-suite/mimics.

[43] Chen J, Wang K, Zhang C, Wang B. An efficient statistical approach to design 3D-printed metamaterials for mimicking mechanical properties of soft biological tissues. Additive Manuf. 2018;24:341–52. 10.1016/j.addma.2018.10.007

